# Supplementary material for: HIVprotI: an integrated web based platform for prediction and design of HIV proteins inhibitors
Source: J Cheminform. 2018 Mar 9;10:12. doi: 10.1186/s13321-018-0266-y (PMC5845081; doi:10.1186/s13321-018-0266-y)
Supplement: Supplementary file 2 — Additional file 2. Source code of HIVProtI web server. [file 13321_2018_266_MOESM2_ESM.zip › HIVprotI_Source-code/hivprotI/weka-3-6-6/doc/weka/gui/explorer/Explorer.CapabilitiesFilterChangeListener.html]

Explorer.CapabilitiesFilterChangeListener


---


|  |  |  |
| --- | --- | --- |
| **Overview** \| **Package** \| **Class** \| **Tree** \| **Deprecated** \| **Index** \| **Help** \| **Weka's home** | |  |
| **PREV CLASS**   **NEXT CLASS** | **FRAMES**    **NO FRAMES**     **All Classes** |  |
| SUMMARY: NESTED \| FIELD \| CONSTR \| METHOD | DETAIL: FIELD \| CONSTR \| METHOD |  |


---


## weka.gui.explorer Interface Explorer.CapabilitiesFilterChangeListener

**All Superinterfaces:**: java.util.EventListener

**All Known Implementing Classes:**: AssociationsPanel, AttributeSelectionPanel, ClassifierPanel, ClustererPanel, PreprocessPanel

**Enclosing class:**: Explorer

---

``` public static interface Explorer.CapabilitiesFilterChangeListener extends java.util.EventListener ```

Interface for classes that listen for filter changes.

**Version:**
:   $Revision: 7193 $

**Author:**
:   FracPete (fracpete at waikato dot ac dot nz)

---

| **Method Summary** | |
| --- | --- |
| `void` | `capabilitiesFilterChanged(Explorer.CapabilitiesFilterChangeEvent e)`             method gets called in case of a change event |

**Method Detail**

### capabilitiesFilterChanged

```
void capabilitiesFilterChanged(Explorer.CapabilitiesFilterChangeEvent e)
```

:   method gets called in case of a change event

    :   **Parameters:**: `e` - the associated change event


---


|  |  |  |
| --- | --- | --- |
| **Overview** \| **Package** \| **Class** \| **Tree** \| **Deprecated** \| **Index** \| **Help** \| **Weka's home** | |  |
| **PREV CLASS**   **NEXT CLASS** | **FRAMES**    **NO FRAMES**     **All Classes** |  |
| SUMMARY: NESTED \| FIELD \| CONSTR \| METHOD | DETAIL: FIELD \| CONSTR \| METHOD |  |


---
